# Sexual behaviors and their correlates among young people in Mauritius: a cross-sectional study

**DOI:** 10.1186/1472-698X-7-8

**Published:** 2007-10-05

**Authors:** Yumiko H Nishimura, Masako Ono-Kihara, Jagdis C Mohith, Renaud NgManSun, Takayuki Homma, Ralph J DiClemente, Delia L Lang, Masahiro Kihara

**Affiliations:** 1 Department of Global Health and Socio-epidemiology, Kyoto University School of Public Health, Yoshida-Konoe-cho, Sakyo-ku, Kyoto-shi, Kyoto, 606-8501, Japan; 2 Mauritius Institute of Health, Powder Mill, Pamplemousses, Mauritius; 3 Indian Ocean Commission, Farquhar Avenue, Quatre Bornes, Mauritius; 4 Division of Pharmaceutical Sciences, Graduate School of Natural Science and Technology, Kanazawa University, Kakuma-machi, Kanazawa, 920-1192, Japan; 5 Department of Behavioral Sciences and Health Education, Rollins School of Public Health of Emory University, 1518 Clifton Road, Atlanta, Georgia 30322, USA

## Abstract

**Background:**

Little is known about the HIV/AIDS epidemic in the Indian Ocean region, including Mauritius. National records suggest a prevalence of HIV in Mauritius of < 1% in the general population, which is one of the lowest prevalence rates in southern Africa. However, HIV-positive cases have been increasing recently in Mauritius. We conducted a cross-sectional survey in January 2003 to assess the prevalence of HIVrelated sexual behaviors and their correlates among young people aged 15–24 years in Mauritius.

**Methods:**

We identified 1200 participants using two-stage cluster sampling. Demographic, social, sexual, and knowledge of HIV/AIDS data were obtained in face-to-face interviews using a structured questionnaire administered by trained interviewers. The prevalence of sexual behaviors was described in relation to gender, and the correlates of ever having had sex and nonuse of condom at last sex were analyzed using logistic regression.

**Results:**

In the target population, 30.9% of males and 9.7% of females reported a history of sexual intercourse. Of the currently sexually active participants, 50.6% of men and 71.2% of women did not use condoms at their last sexual encounter. Logistic regression revealed that work experience and marijuana use were significantly associated with men's sexual experience, whereas being out of school and drinking experience were significantly associated with women's sexual experience. For both men and women, being Christian and visiting nightclubs were associated with having ever had sexual intercourse (P < 0.05). In addition, not using a condom at the first sexual encounter and lack of exposure to a nongovernmental organization (NGO) dealing with HIV/AIDS were associated with the nonuse of condoms at the last sexual encounter (P < 0.05).

**Conclusion:**

Young people in Mauritius are at risk of a future HIV epidemic because behaviors predisposing to HIV infection are prevalent among sexually experienced youth. A focused prevention program targeting young people should be reinforced as part of the National AIDS Control Program, taking into account the predictors of sexual behaviors identified here.

## Background

Mauritius is an island country with a population of 1.2 million, located in the Indian Ocean about 900 km east of Madagascar. The country is noted for its current rapid economic growth and the cultural diversity of its inhabitants, who came from Asia, Africa, and Europe. Little information is available about the status of the HIV/AIDS epidemic on Mauritius and in the Indian Ocean region [1,2].

National records report 374 cumulative HIV/AIDS cases (238 males and 136 females) among Mauritians at the end of 2002 since the first AIDS case was reported in 1987. Of these cases, almost 70% occurred through heterosexual contact and nearly 15% through either heterosexual contact or injecting drug use (IDU), although IDU-related cases have increased rapidly as of 2003. The peak age of reported cases up to 2002 was 25–39 years for men and 20–29 years for women. Based on HIV antibody tests for blood donors and pregnant women, the estimated HIV prevalence in the general population of Mauritius is < 1%, which is one of the lowest prevalence in the African region. However, concerns about an HIV epidemic has been growing in this country because the number of reported HIV/AIDS cases continued to increase during the last decade, and suddenly doubled from 50 cases in 2000 to 98 cases in 2002 [3]. In addition, Mauritius hosts more than 700,000 tourists annually, mainly from Europe, South Africa, and India, where HIV epidemics are more pronounced than in Mauritius. Tourists can potentially transmit HIV and sexually transmitted diseases (STDs) from country to country [4]. Growing number of people from overseas is causing concerns about a potential HIV epidemic in Mauritius.

The government of Mauritius launched the National HIV/AIDS Strategic Plan in 2001 and identified 12 strategic objectives [3]. After a reduction in new STI/HIV infections among groups with high-risk behaviors (sex workers, IDUs, men who have sex with men, and prison inmates), reducing vulnerability among the youth and children was the third strategic objective of the plan. About 17% of reported HIV/AIDS up to 2001 were among those aged 15–24. Although more cases were reported among those aged 25–39 for men and 20–29 for women, actual disease contraction must have occurred when these people were younger because people are typically unaware of their infection for several years. Prevention of HIV/AIDS among young people is thus one of the country's priorities.

Several studies have examined the sexual behavior of young people in Mauritius. One study of female Economic Processing Zone workers in 1994 revealed that 68% of young female workers reported some sort of intimate behavior with their boyfriends, although only 7% reported full penetration (*i*.*e*., penile-vaginal sex) [5]. A nationwide survey of male and female adolescent behavior using two-stage clustered sampling was conducted as part of the Youth Profile of the Republic of Mauritius in 1996. According to the survey, 43% of never-married males and 11% of never-married females aged 18–25 years reported having experienced sexual intercourse, and the mean age at first sexual intercourse was approximately 18 years [6]. Other surveys were undertaken to assess the knowledge, attitudes, behaviors, and practices (KABP) related to HIV/AIDS among individuals aged 15–49 years [7-9].

Although some studies have yielded useful information, they have several limitations in aiding the design of HIV prevention programs for young people. For example, some of these studies targeted subgroups of young people such as female workers or people approached using convenient sampling methods, thus limiting the generalizability of the results. Other studies included populations other than young people, yielding limited information on young people. In addition, the studies assessed the sexual behaviors of people in terms of certain demographic characteristics, and did not adequately assess them in relation to social and cultural factors, although these factors are undoubtedly important determinants of HIV-associated behavior and are important in designing prevention programs [10-12]. Most of the studies were conducted in the mid-1990s; thus, the information needs to be updated because the prevalence of HIV has increased markedly in the ensuing years.

We designed our study to assess the current HIV-related sexual behaviors of unmarried young people and to investigate the correlates of sexual debut and condom nonuse. More broadly, we assessed the possible influence in the lives of young people of changing Mauritian society brought about by modernization and globalization, e.g., educational attainment and exposure to a variety of socializing items, in connection with sexual behavior. Our ultimate objective was to assist the National AIDS Control program of Mauritius to monitor, evaluate, and reformulate its HIV prevention program for young people.

## Methods

### Sampling frame

The target population was never-married men and women aged 15–24 years living on the island of Mauritius. This population consisted of approximately 173,200 people, which was about 15% of the total population of Mauritius in 2000 [13].

A two-stage cluster sampling method was used to select respondents. The enumeration areas (EAs) of the Central Statistics Office were used as the sampling frame for the first stage. Each EA consisted of approximately 300–600 people comprising 75–150 households, and those in the capital city of Port Louis and Plaines Wilhems District were defined as being from urban areas (43% of the total population). In the first stage, 50 EAs from a total of 3472 were selected using the probability proportional to size (PPS) method [14,15]. All never-married persons aged 15–24 years in selected areas were listed during the preparatory fieldwork for a total of 2592 persons. In the second stage, 12 males and 12 females from each EA were selected using a computer-generated random numbers algorithm. Using this multistage procedure, 600 males and 600 females were chosen to participate in the survey. The sample size was chosen to allow us to detect a 10% difference in sexual experience rate between in-school and out-of-school students with sufficient statistical significance (α = 0.05, two-tailed) and a power of 0.8, as well as to adjust for the confounding effect of multiple factors.

Of the young people selected, 59 respondents (4.9%) did not participate. The primary reasons for nonparticipation were "moved away," "gone abroad," "refused (by himself/herself or parents)," and "married." Consequently, 1141 young people (575 men and 566 women) completed the survey interviews, yielding a response rate of 95.1%. Comparisons of participants and nonparticipants showed no marked difference in sociodemographic characteristics with respect to gender, religion, or age.

### Instrument development

The research questionnaire was developed based on the Family Health International (FHI) HIV/AIDS/STD Behavioral Surveillance Surveys (BSS) Questionnaire for youth [15]. This instrument includes questions that can assess internationally agreed-upon indicators for young people's behaviors in relation to HIV/AIDS [16].

A multiphase process was used to develop the research instrument to ensure that it was developmentally, culturally, and linguistically appropriate. First, seven focus group discussions were conducted in youth centers to tailor the instrument culturally and linguistically for Mauritian youth. Second, persons working in nongovernmental organizations (NGOs) that deal with HIV/AIDS and drug use were invited to review the instrument and make recommendations for its modification. Third, the English instrument was translated into Creole and back-translated into English to confirm the accuracy of the Creole translation. Finally, the instrument was pilot-tested with 50 young people during preparatory fieldwork. Fieldworkers reported the items for which participants had difficulty in understanding the phrase or terms of the question after the pretest interview. The questionnaire was modified based on the fieldworkers' comments and finalized in January 2003.

The final version of the instrument included the following domains of interest: sociodemographic characteristics (19 items), social life (15 items), knowledge and attitude about condoms (10 items), sexual experience (22 items), and knowledge of HIV/AIDS and exposure to an HIV/AIDS program (30 items). A complete questionnaire may be obtained from the first author.

The sociodemographic variables included sex, age, religion, educational background, and work experience. Religion represents racial/ethnic background of the participants. Usually, Hindus represent people whose ancestors came from India, Muslims, from India and the Middle East, and Christians, from Africa, Europe, and China. Concerning social life, questions were asked regarding respondents' mobile phone use, nightclub visits, pornographic film viewing, Internet use, and alcohol and other drug use. Items concerning knowledge and attitude about condoms included questions related to obtaining and the ability to use condoms.

In the section on sexual behavior, young people were first asked about lifetime sexual experiences. Sexual intercourse was defined as vaginal or anal penetration. Those who reported a history of sexual intercourse responded to further questions, including the age of first sexual intercourse, condom use at the first sexual encounter, age of the partner, number of lifetime sexual partners, and sexual activity in the previous 12 months. Those who were sexually active in the previous 12 months were also asked questions regarding the use of condoms at the last sexual encounter and the frequency of condom use with both commercial and noncommercial partners. A commercial partner was defined as a partner with whom a respondent had sex in exchange for money; any partner other than this was defined as a noncommercial partner.

The section on HIV/AIDS knowledge contained 11 items, including modes of HIV transmission and measures for preventing sexual transmission. Items on the exposure to HIV/AIDS prevention programs had questions on exposure to posters on HIV/AIDS and knowledge of government programs and NGOs.

### Instrument validation

To examine the instrument's reliability, 45 items extracted from the original survey were retested 1 week later for 48 randomly selected respondents. Overall, a high test-retest correlation was observed between items (Pearson *r *= 0.85–1.00 [median = 0.96] for continuous variables, kappa = 0.26–1.00 [median = 0.71] for categorical variables) [17]. The internal consistency was satisfactory (α = 0.68) for 11 items of knowledge. In addition, a random response technique was administered to all subjects to assess the validity of the interview-based response on sexual experience in a subsequent procedure [18-20]; at the end of interview, respondents were asked to answer one of two "yes/no" questions, (1) "Is your birthday on the 1st to 10th of the month?" or (2) "Have you ever had sexual intercourse?" by randomly picking up a piece of paper that contained one of these questions. The interviewer did not know which question they answered. Under this less intimidating circumstance, 28.4% male and 7.4% female were estimated to have answered "yes" to question 2. These prevalence rates were comparable to those obtained in the face-to-face interviews (30.9% for men and 9.7% for women).

### Data collection

A team of 50 trained fieldworkers under the direct supervision of five superiors conducted the fieldwork. Data were collected over a 3-week period in January and February 2003. Fieldworkers visited the homes of the respondents and interviewed them in their native language, Creole, using the structured questionnaire. Due to the sensitive nature of some questions, the gender of the interviewers was matched to that of the respondents. The interviews took 20–30 min to complete on average. The decision to use face-to-face interviews was made by taking into account the local language environment of Mauritius where the first language (Creole) is a highly conversation-based language, and French or English is not understood by people with a low educational attainment.

### Statistical analysis

Statistical analyses were performed using SPSS Complex Samples 12.0J (SPSS Japan, Tokyo, Japan) and SUDAAN 8.0.2 (Research Triangle Institute, Research Triangle Park, NC, USA) to account for the clustered sampling design. First, we assessed the prevalence of HIV-related sexual behaviors. Subsequently, using "being sexually experienced" and "condom nonuse at last sex" as the outcome variables, bivariate associations with dichotomous correlates were analyzed using cross-tabulation. Finally, variables that achieved a statistically significant association (*P *< 0.10) in the bivariate analyses and variables that were epidemiologically important were entered into logistic regression models to obtain an adjusted odds ratio for each association.

### Ethical issues

The study protocol was reviewed and approved by the Kyoto University Graduate School and Faculty of Medicine Ethics Committee and Mauritius Ministry of Health and Quality of Life. Before each interview, the interviewer explained the objectives and methodology of the study to each participant; the interviewer signed the survey document when verbal informed consent was obtained from the participant.

## Results

### Description of the sample

The mean age for men was 19.2 years (*SE *= 0.14) and that for women was 18.6 years (*SE *= 0.11). The median ages were 19 years for men and 18 years for women, respectively. Religious affiliations comprised approximately 55% Hindu, 20% Muslim, and 25% Christian. Nearly half of the participants were still attending school and lived in urban areas at the time of the survey. For men, 45.8% were employed full time, whereas 57.4% of women had not worked in the previous 12 months (Table 1).

**Table 1:** Demographic characteristics of participants by gender

	Male (*n *= 575) %	Female (*n *= 566) %
Age group		
15–19	53.1	63.3
20–24	46.9	36.7
Mean age	19.2 (*SE *0.14)	18.6 (*SE *0.11)
		
Religion		
Hindu	55.7	52.6
Muslim	20.5	20.6
Christian	22.8	25.8
Other/No religion	1.0	1.0
		
Education status		
Currently attending school	41.2	48.5
Completed > 3 years in secondary	39.6	38.2
Completed ≤ 3 years in secondary	19.2	13.0
Never attended school	0	0.3
		
Geographic region of residence		
Urban	42.8	42.9
Rural	57.2	57.1
		
Work experience in the previous 12 months		
Full time	45.8	29.9
Part time	19.5	12.7
Did not work	34.7	57.4
		
Monthly personal expenditure (in Rupees)^a^		
High	65.4	48.7
Low	34.6	51.3
Mean expenditure	1662.5 (SE 75.68)	1307.8 (SE 93.62)

*SE*, standard error.

a. Median split of total sample spending pattern: "Low," < 1000; "High," ≥ 1000; *n *= 574 for males and *n *= 561 for females.

### Sexual behavior

The proportion of people who had ever had penetrative sexual intercourse differed significantly between men (30.9%; 95% CI, 25.8–36.5) and women (9.7%; 95% CI, 6.5–14.0; *P *= 0.001). The mean age of first sex among those sexually experienced was 17.4 years for males (median = 17) and 17.8 years for females (median = 18). The mean age of the first partner was 21.4 years for females (median = 21), indicating that women tend to have an older first partner. Significantly more men (65.0%) than women (32.0%) reported having had more than one sexual partner (*P *= 0.001). Condom use during sexual intercourse was low for both men and women, and only 35.7% of men and 24.5% of women reported having used a condom consistently with their noncommercial sex partner during the 12 months before completing the survey (Table 2). Only 23 male respondents reported sex with a commercial partner.

**Table 2:** Prevalence of sexual behavior by gender

	Male % (95% CI)	Female % (95% CI)	*P*^d^(*χ *^2^)^e^
Ever had sex^a^	30.9(25.8–36.5)	9.7(6.5–14.0)	0.001 (51.53**)
Mean age at first sexual encounter^b^	17.4 (*SE *0.20)	17.8 (*SE *0.33)	0.362
Mean age of first partner^b^	18.1 (*SE *0.25)	21.4 (*SE *0.58)	0.001
Used condom at first sexual encounter^b^	41.1 (31.9–51.1)	32.0 (18.7–49.0)	0.277 (1.21)
Had multiple sexual partners^b^	65.0 (56.8–72.5)	32.0 (21.5–44.8)	0.001 (14.83**)
Had sex in the past 12 months^b^	71.3 (63.5–78.1)	75.1 (57.4–87.1)	0.688 (0.16)
Used a condom at last sexual encounter^c^	49.4 (38.2–60.6)	28.8 (12.1–54.2)	0.079 (3.23)
Always used a condom^c^	35.7 (25.6–47.4)	24.5 (10.1–48.4)	0.274 (1.23)

CI, confidence interval; *SE*, standard error.

a. Among total respondents (*n *= 575 for males and *n *= 566 for females).

b. Among people who had ever had sex (*n *= 183 for males and *n *= 55 for females).

c. Among people who had sex with noncommercial sex partners in the past 12 months (*n *= 123 for males and *n *= 40 for females).

d. Chi-square test for the percentage and *t*-test for the mean.

e. ** *P *< 0.01.

### Correlates of sexual experience

The correlates of sexual experience in terms of sociodemographic characteristics and social life indicators such as exposure to nightclubs and drug use were analyzed by gender (Table 3). Logistic regression results indicate that for both sexes, non-Christians (i.e., Hindus and Muslims) had a significantly lower adjusted odds ratio (AOR) of being sexually experienced (AOR = 0.27 for men and 0.15 for women). Men who had ever worked, ever visited nightclubs, or had a history of using marijuana had a significantly higher likelihood of being sexually experienced (AOR = 3.14, 3.07, and 3.09, respectively). Although not statistically significant in the logistic regressions, most social life factors among men, i.e., having a mobile phone, a history of watching pornographic films, and drinking alcohol, were associated with being sexually experienced in bivariate analyses. Women who were not in school, had ever visited nightclubs, or reported a history of alcohol use in the 12 months before the survey had a significantly higher likelihood of being sexually experienced in logistic regression analyses (AOR = 4.28, 3.14, and 2.81, respectively).

**Table 3:** Bivariate and multivariate association between dichotomous correlates and ever having had sex (male and female)

Correlate			Male						Female			
	
			Bivariate		Multivariate ^a^	*n *= 575			Bivariate		Multivariate ^a^	*n *= 564
	
	*n*	Percent reporting ever had sex	Prevalence ratio (95% CI)	*P *(*χ *^2^)^b^	Adjusted odds ratio (95% CI)	*P*	*n*	Percent reporting ever had sex	Prevalence ratio (95%CI)	*P *(*χ *^2^)^b^	Adjusted odds ratio (95%CI)	*P*
**Sociodemographic**												
Age group (years)												
Older (20–24)	273	42.5	2.06	0.001	1.50	0.131	206	14.5	2.12	0.013	1.18	0.705
Younger (15–19)	302	20.7	(1.63–2.60)	(43.25**)	(0.88–2.56)		360	6.9	(1.20–3.75)	(6.73*)	(0.49–2.89)	
Geographic area												
Urban	236	30.3	0.97	0.847	0.74	0.195	232	11.4	1.35	0.426	0.80	0.630
Rural	339	31.4	(0.67–1.38)	(0.04)	(0.47–1.17)		334	8.4	(0.64–2.86)	(0.65)	(0.32–1.99)	
Currently in school												
No	337	41.8	2.74	0.001	1.75	0.104	288	15.3	4.08	0.001	4.28	0.001
Yes	238	15.3	(1.82–4.13)	(44.13**)	(0.89–3.47)		276	3.8	(1.93–8.62)	(16.51**)	(1.84–9.97)	
Work experience												
Ever worked	376	41.7	3.92	0.001	3.14	0.003	236	15.9	3.13	0.002	1.17	0.701
Never worked	199	10.6	(2.31–6.67)	(48.48**)	(1.50–6.54)		330	5.1	(1.58–6.22)	(10.31**)	(0.52–2.63)	
Religion												
Non-Christian	442	25.3	0.51	0.001	0.27	0.001	422	3.6	0.13	0.001	0.15	0.001
Christian	133	49.8	(0.39–0.66)	(18.53**)	(0.16–0.46)		144	27.0	(0.07–0.26)	(24.46**)	(0.07–0.34)	
**Social life**												
Have a mobile phone												
Yes	318	36.8	1.55	0.001	1.46	0.119	245	12.6	1.75	0.048	1.53	0.250
No	257	23.8	(1.21–1.98)	(14.26**)	(0.90–2.36)		321	7.2	(0.98–3.12)	(4.13*)	(0.73–3.19)	
Ever been to a nightclub												
Yes	254	51.9	3.59	0.001	3.07	0.001	82	28.5	4.67	0.010	3.14	0.027
No	321	14.4	(2.58–5.01)	(83.69**)	(1.74–5.42)		484	6.1	(2.49–8.78)	(7.30*)	(1.15–8.56)	
Ever watched a pornographic film												
Yes	404	36.5	2.10	0.001	1.50	0.123	75	15.1	1.70	0.220	N/A	N/A
No	171	17.4	(1.50–2.96)	(26.90**)	(0.89–2.50)		491	8.9	(0.82–3.56)	(1.54)		
Ever used the Internet												
Yes	228	27.2	0.82	0.085	1.15	0.652	224	7.5	0.66	0.319	N/A	N/A
No	347	33.2	(0.66–1.02)	(3.10)	(0.62–2.13)		342	11.2	(0.28–1.59)	(1.02)		
Ever drank alcohol in the past 12 months												
Yes	252	45.4	2.39	0.001	1.56	0.076	130	23.9	4.38	0.001	2.81	0.014
No	323	19.0	(1.90–3.01)	(58.71**)	(0.95–2.54)		436	5.5	(2.42–7.93)	(12.23**)	(1.24–6.37)	
Ever used marijuana												
Yes	64	68.9	2.59	0.001	3.09	0.001	5	48.8	5.25	0.124	N/A	N/A
No	511	26.6	(1.99–3.37)	(24.09**)	(1.73–5.51)		561	9.3	(1.97–13.98)	(2.46)		

N/A, not applicable; 95% CI, 95% confidence interval.

a. Three basic sociodemographic variables (age group, geographic area, religion) and variables that attained *P *< 0.10 in bivariate analyses were entered into the logistic regression model.

b. * *P *< 0.05. **P < 0.01.

### Correlates of condom nonuse at the last sexual encounter

Of those who had sex with noncommercial partners in the last 12 months (*n *= 123 for men and *n *= 40 for women), more than 50% of men and 70% of women did not use a condom at the last sexual encounter. The correlates of condom nonuse at the last sexual encounter were analyzed in terms of sociodemographic characteristics, condom attitudes, sexual behaviors, HIV/AIDS knowledge, and exposure to an HIV/AIDS program (Table 4). Male and female data were combined for condom nonuse analyses because prior bivariate analyses stratified by gender indicated that the direction and magnitude of all independent variables were similar for men and women. In the logistic regression analyses, condom nonuse at the first sexual encounter was found to be the most significant correlate of nonuse at the last sexual encounter (AOR = 12.92). Moreover, those who had not been exposed to HIV prevention programs by NGOs that promoted HIV prevention among young people had a significantly higher AOR (3.27). Those who were female and older had higher likelihoods (AOR = 3.09 and 2.68, respectively) of not using a condom at the last sexual encounter, although these factors were not statistically significant in logistic regression analyses.

**Table 4:** Bivariate and multivariate association between dichotomous correlates and engaging in sex without a condom at the last sexual encounter with a noncommercial partner

Correlate			Bivariate		Multivariate ^a^	*n *= 161
	
	n	Percent reporting condom nonuse at last sexual encounter	Prevalence ratio (95% CI)	*P *(*χ *^2^)^b^	Adjusted odds ratio (95% CI)	*P*
**Sociodemographic**						
Gender						
Female	40	71.2	1.41	0.079	3.09	0.056
Male	123	50.6	(0.94–2.11)	(3.23)	(0.97–9.85)	
Age group (years)						
Older (20–24)	104	61.6	1.45	0.034	2.68	0.051
Younger (15–19)	59	42.5	(1.00–2.11)	(4.78*)	(1.00–7.23)	
Geographic area						
Urban	66	45.0	0.73	0.065	0.95	0.893
Rural	97	62.0	(0.53–0.99)	(3.56)	(0.43–2.11)	
Currently in school						
No	127	58.1	1.42	0.051	0.99	0.984
Yes	36	40.9	(0.97–2.09)	(4.02)	(0.27–3.56)	
Work experience						
Ever worked	132	55.8	1.16	0.520	N/A	N/A
Never worked	31	48.2	(0.74–1.81)	(0.42)		
						
**Condom attitudes**						
Perceived difficulty in obtaining a condom						
High	23	80.4	1.58	0.017	4.17	0.084
Low	138	50.9	(1.18–2.12)	(6.17*)	(0.82–21.14)	
Self-efficacy of using a condom with a new partner						
Low	28	88.5	1.83	0.001	3.11	0.066
High	133	48.3	(1.46–2.30)	(27.38**)	(0.93–10.41)	
Confidence to refuse sex without condom						
Low	86	60.4	1.28	0.111	N/A	N/A
High	75	47.2	(0.95–1.72)	(2.64)		
						
**Sexual behaviors**						
Used a condom at first sexual encounter						
No	103	78.3	3.75	0.001	12.92	0.001
Yes	60	20.9	(2.34–6.01)	(38.64**)	(4.96–33.62)	
Had more than one sexual partner						
Yes	111	54.4	0.99	0.958	N/A	N/A
No	52	54.9	(0.74–1.34)	(0.00)		
						
**Knowledge of HIV prevention methods**						
Effectiveness of condom in HIV prevention						
Low	20	79.1	1.54	0.029	1.94	0.398
High	143	51.4	(1.13–2.10)	(5.06*)	(0.41–9.19)	
Effectiveness of having one faithful partner in HIV prevention						
Low	60	58.7	1.13	0.454	N/A	N/A
High	103	52.0	(0.83–1.54)	(0.57)		
						
**Exposure to HIV program**						
Ever heard of PILS (NGO)						
No	66	66.0	1.42	0.018	3.27	0.010
Yes	97	46.5	(1.08–1.86)	(5.98*)	(1.35–7.90)	

N/A, not applicable; 95% CI, 95% confidence interval; NGO, nongovernmental organization.

a. Three basic sociodemographic variables (gender, age group, and geographic area) and variables that attained *P *< 0.10 in the bivariate analyses were entered into the logistic regression model.

b. * *P *< 0.05. **P < 0.01.

## Discussion

We described the results of a survey on HIV-related sexual behaviors among a population-based probability sample of young people on the island of Mauritius. Among unmarried young people aged 15–24 years, 30.9% of males and 9.7% of females reported a history of penetrative sexual intercourse. Of these sexually active people, behaviors that increase vulnerability to HIV infection were prevalent, e.g., having multiple partners (males), having sex without a condom, and having an older partner (females) [21-23].

The proportion of sexually active unmarried youth in Mauritius, i.e., 30.9% of men and 9.7% of women, appears to be relatively low compared to that in other African countries. Data from 17 national surveys conducted in the past 5 years among young people aged 15–24 years from diverse countries in sub-Saharan Africa indicate that, on average, about 38% of men and 30% of women report having had premarital sex in the previous year [24]. As prevalence of ever having had sex in these countries should be higher than that of sex in previous year, we can say that sexual activity of Mauritian youth is comparatively much lower than that of other African countries. In addition, when compared with data from the 1996 Mauritius Youth profile study, we did not observe a dramatic change in the proportion of sexually experienced unmarried youth. This relatively low prevalence of sexually active youth may be due at least in part to the predominance of religions with conservative sex norms and may partly explain how Mauritius has managed to maintain a stable low level of HIV infection in the 1990s, despite the devastating HIV epidemic in neighboring Africa.

Nevertheless, these findings do not leave room for complacency. Among sexually experienced Mauritian youths, 50.6% of male and 71.2% of female respondents did not use condoms at the last sexual encounter with a noncommercial partner. Although HIV infection risks may not be very high for young people as a whole, there certainly exist a group of youth who have unprotected sex and are vulnerable to HIV infection. Therefore, the HIV program for youth in Mauritius should aim to maintain the low prevalence of sexually active youth and to promote safe sexual behavior among those who are sexually active.

To achieve these goals, we need to identify the predictors of sexual behavior to effectively and efficiently target HIV prevention to this vulnerable subpopulation. We identified several factors related to the sexual behaviors of young people in Mauritius. For men, having ever worked and marijuana use were significantly associated with having ever had sex. In contrast, being out of school and drinking were associated with women's sexual experience. In addition, being Christian and visiting nightclubs were significantly associated with sexual experience in both sexes. Furthermore, among sexually experienced young people, the use of condom at first sex and exposure to an NGO's HIV program were significantly related to condom use at last sexual encounter. These findings suggest possible directions for effective youth HIV prevention programs in Mauritius. We grouped them into three possible approaches: (1) out-of school and in-school approaches, (2) nightclub and popular site approaches, and (3) religion and social status approaches.

### Out-of-school and in-school approaches

Prevention efforts are needed outside the school system because the young people that were more sexually experienced were men who have ever worked or women who are not in school. Two channels currently exist in Mauritius to reach young people outside the school system: (1) peer educators trained by the AIDS unit of the Ministry of Health and (2) youth leaders attached to the youth centers of the Ministry of Youth and Sports. These resources should be reinforced to reach out-of-school youth to provide better reproductive health services. Furthermore, one of our most important findings was that exposure to an NGO program was associated with safer sexual behavior in young people. Because these activities are easily modifiable, a great opportunity may exist for the promotion of safer sex by reinforcing the activities of NGOs through peer educators or youth leaders.

At the same time, to maintain or reduce the number of out-of-school youth, enrollment in secondary and tertiary education should be promoted in a long-term perspective. In Mauritius, the enrollment ratio for primary school is 100%, but falls to 60% for secondary school, and 4.4% for post-secondary school [25]; there is therefore room to promote secondary and tertiary education. By doing so, young people may delay their sexual debut by having greater access to information and skills, enhanced future orientation and aspirations, and by delaying exposure to the workplace where they encounter older people.

Reproductive health education needs to be promoted in schools because it is important for all young people, including the sexually inexperienced, to have the requisite knowledge and skills to use condoms beginning with their first sexual encounter. Our results indicate that condom nonuse at the last sexual encounter is significantly associated with condom nonuse at the first sexual encounter, i.e., those who used a condom at their first sexual encounter were much more likely to report using a condom at their most recent sexual encounter. The same trends were found among adolescents from other parts of the world [26,27]. These results suggest that HIV prevention programs emphasizing appropriate and consistent condom use should target young people before the onset of sexual activity. In schools, where most students are sexually inexperienced, information on the risks accompanying sexual behavior should be provided to all students so that they can make informed future decisions.

### Nightclub and popular site approaches

Nightclub visitation was correlated with other variables assessing popular sensationseeking behavior, such as possession of a mobile phone, watching pornographic films, alcohol intake, and marijuana use, in bivariate analysis. Although only nightclub visitation was significantly associated with sexual behavior in both men and women in multivariate analyses, the effects of these other items should be considered in the HIV prevention program. Media campaigns using various channels are potentially one of the most effective approaches to convey HIV prevention messages to these young people who seek out and are exposed to risks. As suggested in previous studies, role model stories printed in youth-attracting flyers may be a useful means to convey HIV prevention messages, even to hard-to-reach youths, because they can readily be distributed at popular sites where young people congregate [28,29].

In addition, HIV prevention interventions, including education on the risks of drinking and drug use, could be explored in nightclubs because we identified nightclubs as an important place where sexually experienced young men and women congregate. The identification of specific access points will help the national AIDS control program to effectively use available resources for targeted interventions. Ford and Inman [30] documented successful interventions to promote "safer sex" among young people in nightclubs in southwest England. The event was organized in a way that appeals to young people's sense of humor: competitions run by disc jockeys, free condoms, and "safer sex" materials. Although gaining the cooperation of venue owners may be challenging, such events could be considered in nightclubs in Mauritius to effectively reach young people with high risk factors for contracting HIV.

The effect of watching pornographic films should also be cautiously assessed because the bivariate analysis, but not logistic regression, showed this to be significantly associated with sexual experiences in young men. In fact, 70.6% of men, as compared to 12.9% of women, have reported ever having watched pornographic films. These films, most of which are from overseas, include unprotected sexual behaviors. A recent cohort study in the United States indicated that exposure to sexual content in the media accelerated adolescents' sexual activity and increased their risk of engaging in early sexual intercourse [31]. Similarly, a study in China reported that younger students are more exposed to pornographic media than are older students [32]. Taking these findings and the recent rapid increase in Internet use in Mauritius into account, attention should be given to trends related to the excessive exposure of young men to sexual media content. Video rental stores, of which there are many on the island, are potential intervention sites to address the watchers of pornographic films.

### Religion and social status approaches

Christian youth reported more sexual experience than those in other religious groups. This may be partly related to their higher exposure to nightclubs, alcohol, and marijuana use because a relatively higher proportion of Christians live in urban areas compared to Muslims or Hindus. In this context, the popular site approaches (above) should be implemented, focusing on Christian youth.

Given disproportionate differences in sexual experience rates among religious groups, religious gatherings should also be considered as potential places for HIV prevention sessions to address sexual norms and reproductive health [33]. Religious groups in Mauritius are still unwilling to promote condoms as a means of protection, and would rather stress abstinence and fidelity. Some religious people, however, are more involved in the fight against poverty, the commercial sex trade, and substance abuse, and through these related issues, they also deal with HIV/AIDS. Greater participation of religious leaders in HIV/AIDS programs may be facilitated through greater involvement of people living with HIV/AIDS (PLWHA), as suggested by work in Trinidad, where the religious profile is as diverse as in Mauritius [34]. In Trinidad, personal interaction with PLWHA was positively associated with the involvement of religious leaders in HIV/AIDS initiatives.

Finally, it is needless to say that National AIDS Control program of Mauritius should swiftly act on HIV prevention for injecting drug users given the contemporary trends of HIV spread in this group. At the same time, with regard to young people, our findings suggest that early and focused programs are needed. Evidence from Cambodia indicates that young people are more willing to adopt safer behaviors than older people when provided with the appropriate services [35]. Focused programs specifically targeting young people in general, as well as at-risk populations, such as sex workers and drug users, will have maximum impact and require the least resources when implemented at this early stage of the HIV epidemic. In particular, young women warrant special attention because the results indicate that women tended to have older sexual partners, which means that they may be more exposed to HIV than men [22]. In Africa, young people, especially younger women, are the most seriously affected by HIV because of their social and biological vulnerability [36]. Precautions are needed to protect younger women in Mauritius to avoid a situation similar to that on the African continent. The National AIDS Control Program of Mauritius should take every opportunity to intervene before the HIV epidemic escalates.

Our study is not without limitations, which include face-to-face interviews as the only modality of data collection. Although under-reporting might not have been completely avoided, the validity of the responses concerning sexual experience was assessed using a random response technique and considerable effort was made to put the respondents at ease, particularly for sensitive questions. However, self-report information may be subject to reporting errors and biases. Furthermore, the study was based on cross-sectional data for which the direction of causal relationships cannot be determined. Future studies need to assess the effectiveness of HIV prevention interventions in the context of young people's lives in Mauritius.

## Conclusion

Premarital sexual intercourse was reported by 30.9% of male and 9.7% of female young people in Mauritius. Of these sexually active young people, behaviors that increase the vulnerability to HIV infection were prevalent and were associated not only with their culture, which was represented by religion, but also with the influence of a changing society such as school enrolment, NGOs, nightclubs, and pornographic films. Taking these predictors of sexual behaviors into account, a focused HIV prevention program for young people needs to be reinforced in Mauritius to minimize the future consequences of the epidemic, even though HIV levels are currently low.

## Competing interests

The author(s) declare that they have no competing interests.

## Authors' contributions

YHN, MOK, and MK conceived the study, performed and interpreted the analyses, and wrote the first draft of the manuscript. JCM and RN supervised and coordinated the data collection in the field. TH, RJD, and DLL provided advice on the statistical data analysis and interpretation of results. All authors read and approved the final manuscript.

## Pre-publication history

The pre-publication history for this paper can be accessed here:



## References

[B1] Matteelli A, Pizzocolo C, Bakar HA, Toyb M, Pyndiah N, Udonwa NE, Gedeon AG, Farina C, Caligaris S, Castelli F, Carosi G (2002). STI epidemics in the Indian Ocean region: can the phase be assessed?. Sex Transm Infect.

[B2] Andriamahenina R, Ravelojaona B, Rarivoharilala E, Ravaoarimalala C, Andriamiadana J, Andriamahefazafy B, May JF, Behets F, Rasamindrakotroka A (1998). [AIDS in Madagascar. I. Epidemiology, projections, socioeconomic impact, interventions]. Bull Soc Pathol Exot.

[B3] Ministry of Health and Quality of Life (2001). National HIV/AIDS Strategic Plan 2001-2005, Mauritius.

[B4] Bellis MA, Hughes K, Thomson R, Bennett A (2004). Sexual behaviour of young people in international tourist resorts. Sex Transm Infect.

[B5] Schensul SL, Schensul JJ, Oodit G, Bhowon U, Ragobur S (1994). Sexual intimacy and changing lifestyles in an era of AIDS: Young women workers in Mauritius. Reproductive Health Matters.

[B6] Mauritius Institute of Health (1996). Study on youth profile in the republic of Mauritius.

[B7] Mauritius Institute of Health (1998). Report on knowledge, attitudes, beliefs and practices related to HIV/AIDS in republic of Mauritius.

[B8] Ministry of Health, University of Mauritius, WHO (1989). Knowledge attitudes beliefs practices related to AIDS, KABP survey 1989 Mauritius.

[B9] Ministry of Health, University of Mauritius, WHO, UNICEF (1992). Rodrigues STD/AIDS Knowledge attitudes beliefs practices: a qualitative KABP study.

[B10] Crosby RA, Holtgrave DR, DiClemente RJ, Wingood GM, Gayle JA (2003). Social capital as a predictor of adolescents' sexual risk behavior: a state-level exploratory study. AIDS Behav.

[B11] DiClemente RJ, Wingood GM, Crosby R, Cobb BK, Harrington K, Davies SL (2001). Parent-adolescent communication and sexual risk behaviors among African American adolescent females. Journal of Pediatrics.

[B12] Ono-Kihara M, Kihara M, Yamazaki H (2002). Sexual practice and the risk for HIV/STDs infection of youth in Japan. Japan Medical Association Journal.

[B13] Central Statistics Office, Ministry of Economic Development Financial Services and Corporate Affairs (2001). 2000 Housing and population census Republic of Mauritius, Volume II: Demographic and fertility characteristics.

[B14] Bennett S, Woods T, Liyanage WM, Smith DL (1991). A simplified general method for cluster-sample surveys of health in developing countries. World Health Statistics Quarterly.

[B15] Family Health International (2000). Behavioral Surveillance Surveys BSS Guidelines for repeated behavioral surveys in populations at risk of HIV.

[B16] UNAIDS (2000). National AIDS programmes, A guide to monitoring and evaluation.

[B17] Brener ND, Kann L, McManus T, Kinchen SA, Sundberg EC, Ross JG (2002). Reliability of 1999 Youth Risk Behavior Survey Questionnaire. Journal of Adolescent Health.

[B18] Dare OO, Cleland JG (1994). Reliability and validity of survey data on sexual behaviour. Health Transit Rev.

[B19] Fisher M, Kupferman LB, Lesser M (1992). Substance Use in a School-Based Clinic Population: Use of the Randomized Response Technique to Estimate Prevalence. Journal of Adolescent Health.

[B20] Kolata G (1987). How to ask about sex and get honest answers. Science.

[B21] Ford K, Lepkowski JM (2004). Characteristics of sexual partners and STD infection among American adolescents. Int J STD AIDS.

[B22] Miller KS, Clark LF, Moore JS (1997). Sexual initiation with older male partners and subsequent HIV risk behavior among female adolescents. Fam Plann Perspect.

[B23] Vanoss Marin B, Coyle KK, Gomez CA, Carvajal SC, Kirby DB (2000). Older boyfriends and girlfriends increase risk of sexual initiation in young adolescents. J Adolesc Health.

[B24] USAID, UNICEF, CDC, UNAIDS, WHO, US Census Bereau, Family Health International, MEASURE Evaluation, The Synergy Project, MEASURE DHS+ (2006). HIV/AIDS Survey Indicators Database. http://www.measuredhs.com/hivdata/.

[B25] Central statistics office, Ministry of Economic Development Financial Services and cooperate Affairs (2000). Mauritius in figures 2000.

[B26] St.Lawrence JS (1993). African-American Adolescents' Knowledge, Health-Related Attitudes, Sexual Behavior, and Contraceptive Decisions: Implications for the Prevention of Adolescent HIV Infection. Journal of Consulting and Clinical Psychology.

[B27] Svare EI, Kjaer SK, Poll P, Bock JE (1997). Determinants for contraceptive use in young, single, Danish women from the general population. Contraception.

[B28] Bond L, Bowden-Proctor J, Lauby J, Walls C, Woll M (1997). Developing non-traditional print media for HIV prevention: role model stories for young urban women. Am J Public Health.

[B29] Corby NH, Enguidanos SM, Kay LS (1996). Development and use of role model stories in a community level HIV risk reduction intervention. Public Health Rep.

[B30] Ford N, Inman M (1992). Safer sex in tourist resorts. World Health Forum.

[B31] Brown JD, L'Engle KL, Pardun CJ, Guo G, Kenneavy K, Jackson C (2006). Sexy media matter: exposure to sexual content in music, movies, television, and magazines predicts black and white adolescents' sexual behavior. Pediatrics.

[B32] Ma Q, Ono-Kihara M, Cong L, Xu G, Zamani S, Ravari SM, Kihara M (2006). Sexual behavior and awareness of Chinese university students in transition with implied risk of sexually transmitted diseases and HIV infection: a cross-sectional study. BMC Public Health.

[B33] Agadjanian V (2005). Gender, religious involvement, and HIV/AIDS prevention in Mozambique. Soc Sci Med.

[B34] Genrich GL, Brathwaite BA (2005). Response of religious groups to HIV/AIDS as a sexually transmitted infection in Trinidad. BMC Public Health.

[B35] MAP Monitoring the AIDS pandemic (2004). AIDS in Asia: Face and Facts: A comprehensive analysis of the AIDS epidemics in Asia.

[B36] UNAIDS, WHO (2004). AIDS Epidemic Update.

